# Prevalence, associated factors and consequences of substance use among health and medical science students of Haramaya University, eastern Ethiopia, 2018: a cross-sectional study

**DOI:** 10.1186/s12888-019-2340-z

**Published:** 2019-11-06

**Authors:** Wubet Alebachew, Agumasie Semahegn, Tilahun Ali, Hailemariam Mekonnen

**Affiliations:** 1 College of Health Sciences, Debre Tabor University, P.O.Box:272, Debre Tabor, Ethiopia; 2 College of Health and Medical Sciences, Haramaya University, P.O.BOX 235, Harar, Ethiopia; 3 College of Health and Medical science, Bahir Dar University, P.O.BOX 79, Bahir Dar, Ethiopia

**Keywords:** Khat chewing, Cigarette smoking, Alcohol drinking, Substance use, Haramaya University

## Abstract

**Background:**

Substance use has a terrible impact on health, behavior and country’s economy because the number of people particularly the youngsters being involved in this practice is increasing rapidly. However, the prevalence, determinants and consequence of substance use in the study area has been overlooked.

**Methods:**

A descriptive quantitative cross-sectional study was conducted among 254 health science students of Haramaya University. The respondents were selected randomly after double stratification based on their department and batch respectively. A pre-tested self–administrable anonymous questionnaire was used. The collected data were entered into epidata version 3.1 and exported to SPSS version 23. Descriptive statistical analysis was done to examine findings. Besides, chi-square (X^**2**^) test was considered to examine the nonparametric association of factors with ever substance use.

**Results:**

Prevalence of ever substance use for at least one substance was found to be 114(45.4%). Ever khat chewers take the highest percentage [107(93.9%)] followed by ever smokers 45(39.5%) and ever drinkers 44(38.6%). Among these ever substance users, 80(70.2%) were found to be current substance users. Being a preparatory student (26.3%) and freshman at university (57.9%) were critical times to initiate substance use. Sex, monthly income, sexual risk behavior and family history of substance use were found to be significantly associated with being ever substance user as witnessed by their respective X^**2**^ values of 19.67, 72.28, 28.99 and 139.72 at *P*-value = 0.05 and degree of freedom (d_f_ = 1). From the overall ever substance users, 31.6% had undesirable health consequences. Among these consequences, anorexia [40 (35.1%)] accounted for the highest percentage followed by insomnia [29 (25.4%)], depression [25 (22%)], gastritis [25 (22%)], dental caries [23 (20.2%)] and increased sexual activity [12 (10.5%)].

**Conclusion:**

Prevalence of ever substance use in the study area was relatively high. Therefore attention should be given to the major reasons for substance use mainly orientation of freshman students about better stress coping mechanisms and expansion of adequate recreational activities.

## Background

Substance use has become one of the rising major public health and socio-economic problems worldwide. Hard drugs like cocaine are rarely available in Ethiopia where as Khat, alcohol and cigarette are commonly available and used substances [1]. Khat is originated from Ethiopia, especially in Hararghe region with the gradual expansion to the different parts of the country and other nations in Africa and Arabia [1–6]. According to the world health organization (WHO) estimation, approximately 47% of men and 12% of women smoke cigarette worldwide in 2010.The WHO regards smoking as pandemic while attributing more than 4 million deaths in a year to tobacco and it is expected that this figure will rise to 10 million deaths by the year 2020. More people smoke today than any other time in human history. One person dies every 10 seconds due to smoking-related diseases [7]. Smoking is practiced together with khat chewing and drinking alcoholic beverages that have many consequences [8]. Annually, in the United States, about half million people die of different diseases attributable to cigarette smoking. Thus, nearly 6 million years of potential life loss, $82 billion economic mess and $ 75 billion direct medical expenses were reported from the country. Furthermore, cigarette smoking has been considered as “an entry point” towards forbidden drug use among adolescents [9].Globally, 9% of the major non-communicable diseases and 71% of lung cancer deaths are attributed to tobacco alone. These major noncommunicable diseases include atherosclerotic heart disease, myocardial infarction, heart failure, malignancy and diabetic mellitus that have paramount association and causal linkage with oral consumption of tobacco. Besides, socio-economic factors like education and occupational status were considered to be possible risk factors of these chronic non-communicable diseases. Therefore, practical consideration of efforts to modify these factors can bring a multitude of positive outcomes [10–13]. Alcohol drinking is spreading in universities and other tertiary academic institutions. In terms of gender, though men appear to drink more, women are also increasingly taking on this habit. Households are spending quality time in drinking and less on agricultural production [2]. Existing literature on alcohol consumption among adolescents in sub-Saharan Africa suggests that a substantial proportion of adolescents have consumed or currently consume alcohol [3, 14] and alcohol use is generally believed to be the most important for sexual risk behavior in HIV/AIDS transmission because those addicted substances cause behavioral addictions like sex addictions resulting in important social and medical consequences [3]. High risk sexual behavior under the influence of alcohol is common in teenagers because alcohol is thought to fuel HIV transmission by blunting one’s behavioral self-monitoring and increasing the likelihood of multiple sexual partners, unprotected sex, intergenerational sex and commercial sex [15–23]. Some studies have indicated that substance use resulted in psychological stress, suicidal attempts, functional impairment, physical ill health, and risk-taking behavior [8–10, 24–27].Substance use is influenced by different factors, for example, a cross-sectional study held at Harar town, Eastern Ethiopia, revealed that the use of khat was significantly associated with age, gender, Muslim religion, peer influence, and habit of family and other relatives among students [4, 5]. One of the targets in the health goal of the sustainable development goals (SDGs) is to strengthen the prevention and treatment of substance use, including narcotic drug use and harmful use of alcohol. However, in the study area, there has been no prior study about the significance and associated factors of the problem since the launch of SDGs by 2015. Therefore, this study will help to show public health importance of the problem in response to the different preventive and therapeutic measures that have been taken into action since 2015.

## Methods

### Study area and period

This study has been conducted from January 16–30, 2018 at Haramaya University. Haramaya University was established in 1954 next to Addis Ababa University in Ethiopia. It is located about 510 km away from Addis Ababa in Oromia national regional state, East Hararghe administrative zone. The study programs of the university range from undergraduate (Diploma and first degree) to post graduate levels (MSc, Ph.D.) in its 3 campuses and 13 colleges. Among these colleges, College of Health and Medical Science is one of the colleges which was established in September 1996 with the objective of training health professionals who contribute to filling the gap of health need of the country, especially the rural population. It had 8 training programs: Medicine, Pharmacy, Comprehensive Nursing, Public Health, psychiatric Nursing, Midwifery, Medical laboratory technician and Environmental health science.

### Study design and participants characteristics

A descriptive institution based quantitative cross-sectional study design was conducted to determine the prevalence, associated factors and consequences of substance use among students of the college of health and medical science at Haramaya University. All regular health and medical science students of the college were included for the study. Students who were critically ill (unable to read and write) and physically impaired (had a hearing and speaking problem) at the time of data collection were excluded.

### Sample size determination and sampling procedure

**Fig. 1:**
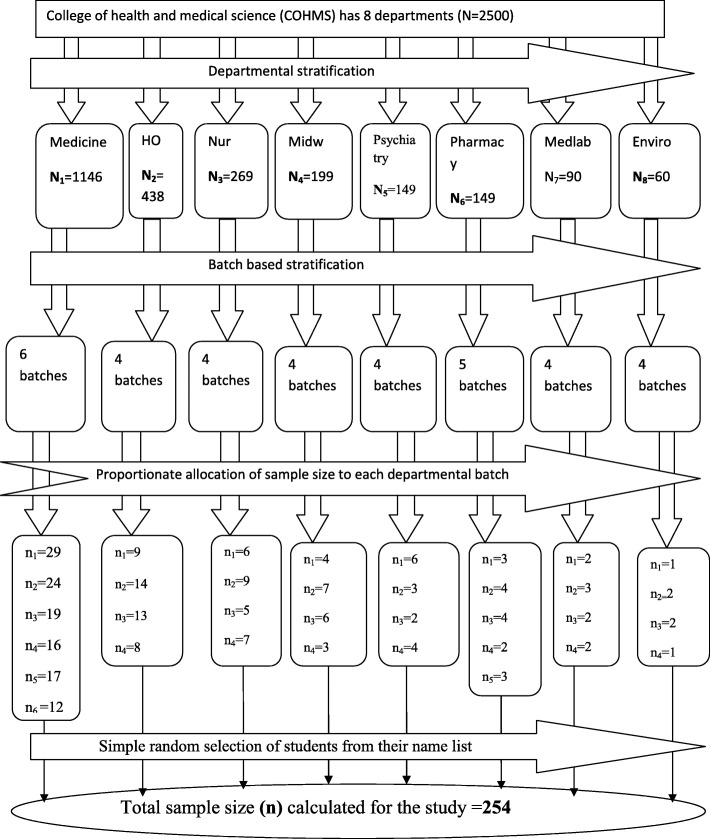
A flow diagram illustrating sampling procedure to reach the calculated 254 sample of students out of 2500 students in the college of health and medical science, HU, 2018

The sample size (n) required for this study was determined using single population proportion formula (*n* = (Zα/2)^2^ p (1-p)/d^2^)); where n = the required minimum and feasible sample size, Za/2(1.96): significance level at α =0.05 with 95% confidence interval, p: proportion of substance use in Mekelle University (21%) [18], and d: margin of error (5%). The calculated sample size was 255. However, the study population (*N* = 2500) was less than 10,000 and hence the correction formula was used to calculate the exact sample size (n_f_). Therefore, the final required sample size was 254 by considering a non-response rate of 10%. Stratified sampling method followed by simple random technique was used to select individual students that were to be included in the sample. Using a stratified sampling method, the total number of students in the college was first divided into different strata based on their departments. Then each department students were again grouped into other different strata depending on class year (batch). Finally, a simple random sampling technique was used to sample students from each departmental batch by using a student’s rosters as a sampling frame. Eligible students were directly approached by the data collectors while they were at their respective classes in morning time immediately after class attendance. The flow diagram to illustrate how 254 eligible students were approached out of 2500 is described in Fig. 1.

### Measurement and data collection procedure

Data were collected using a structured self-administrable anonymous questionnaire prepared in English that contained open and closed-ended questions which included information of socio-demographic characteristics, prevalence, associated factors and health consequences of substance use. Data were collected by 8 BSC Nurses and supervised by 2 MSc health officers.

### Data quality control

The data quality was assured by different methods. A structured questionnaire adapted from WHO students’ drug use survey questionnaire [22, 23] based on own specific objectives was used (Additional file 1). The questionnaire was validated by pretesting upon 13 (5% of the sample size) sample health science students of other nearby university (Jigjiga University). Two days of training was provided to the data collectors and supervisors on the data collection tool and the data collection procedures. Findings from the pretesting were utilized for modifying and adjustment of the instrument. For the daily activities, data collectors were supervised closely by the supervisors and the principal investigator. Completeness of each questionnaire was checked by the principal investigator and the supervisors on a daily basis.

### Operational definition

#### Ever substance user

A respondent who used the substance even once in his /her life [1, 6, 18, 24, 25, 27].

#### Current substance user

A respondent who used substance at least once in the past most recent 30 days [1, 2, 6, 18].

### Data processing and analysis

After collection of the data, each questionnaire was thoroughly reviewed for completeness and consistency by the data collectors, supervisor and principal investigator. Then, the data were coded and entered into Epi data version 3.1 and analyzed by using SPSS for window version 23. Descriptive statistical analysis was used to examine findings of the study using frequency, mean, and proportion. The results were presented using figures, tables and text form. Bivariable analysis was carried out using the chi-square test to examine the association between substance use and associated factors. In doing so, a *P*-value of 0.05 or less was used as cut off level for statistical significance at degree of freedom = 1.

## Results

### Socio-demographic characteristics of respondents

Though it was aimed to collect data on the calculated 254 sample of eligible students, 3 students (2 medical and 1 Pharmacy) didn’t answer more than half of the questions in their respective self administered questionnaires making a response rate of 98.8%. Majority of the respondents, 171 (68.2%), were males and the mean age was 23 years. Most of the respondents, 95 (37.9%), were orthodox believers followed by Muslim believers, 80 (31.9%). Medical students accounted for the highest proportion of the respondents [115(45.8%)] followed by public health [44 (17.5%)] and Nursing [27(10.8%)] students. Based on batch (class year), third-year students accounted for the highest percentage [81(32.3%)] followed by second-year [57 (22.7%)] and first year [52 (20.7%)] students (Table 1).

**Table 1:** Socio demographic characteristics of the respondents, COHMS, HU, Harar, Ethiopia, 2018 (*n* = 251)

Variable		Population	
		F	%
Sex	Male	171	68.1
	Female	80	31.9
	Total	251	100
Age	18–22	38	15.1
	22–26	213	84.9
	Total	251	100
Religion	Orthodox	95	37.9
	Muslim	85	33.9
	Protestant	45	17.9
	Catholic	13	5.2
	Other	13	5.2
	Total	251	100
Ethnicity	Oromo	70	27.9
	Amhara	55	21.9
	Guraghe	40	15.9
	Tigray	38	1.5
	Somalie	32	12.7
	Aderie	7	2.8
	Other	9	3.6
	Total	251	100
Department	Medicine	115	45.8
	Ho	44	17.5
	Nursing	27	10.8
	Midwifery	20	8.0
	Pharmacy	15	6.0
	Psychiatry	15	6.0
	MLT	9	3.6
	Environmental health science	6	2.4
	Total	251	100
Class year	1st	52	20.7
	2nd	57	22.7
	3rd	81	32.3
	4th	40	15.9
	5th	15	6.0
	6th	6	2.4
	Total	251	100

### Substance use behavior

From the 251 respondents, 114(45.4%) were ever substance users of whom 94(82.5%) were males. Out of these 114 ever substance users, the most majority were from the department of medicine [54 (47.4%)] and public health 20 (17.5%). Ever khat chewers take the highest percentage [107(93.9%)] of substance use. Furthermore, males were found to be more ever Khat chewer [92(86.0%)], ever smoker [43(95.6%)] and ever drinker [36(81.8%)] than females (Table 2). Sixty-eight (63.6%) of the ever chewers were practicing coffee drinking whereas 40(37.4%) of them cigarette smoking and 22(19.3%) alcohol drinking along with their chewing habit. Other substances (hashish, cocaine, cannabis, etc.) were asked but none of these substances were reported to be used.

**Table 2:** Frequency distribution of ever substance users among the respondents, COHMS, HU, Harar, Ethiopia, 2018 (*n* = 114)

Sex	Ever substance user							
	Ever chewer		Ever smoker		Ever drinker		Total	
	F	%	F	%	F	%	F	%
Male	92	86.0	43	95.6	36	81.8	172	87.3
Female	15	14.0	2	4.4	8	18.2	25	12.7
Total	107	100	45	100	44	100	197	100

Among 114 ever substance users, 80(70.2%) were found to be current substance users and 72 (87.8%) of whom were males. Among the total of 80 current substance users, current chewers account for the highest percentage [75(93.8%)] (Table 3). Most respondents [146 (86.4%)] were able to easily access and use the substance of their choice even in their dormitory rooms.

**Table 3:** Frequency distribution of current substance users among the respondents, COHMS, HU, Harar, Ethiopia, 2018

Sex	Current substance users (*n* = 80)							
	Current chewer		Current smoker		Current drinker		Total	
	F	%	F	%	F	%	F	%
Male	69	92	40	100	17	85	126	93.3
Female	6	8	0	0	3	15	9	6.7
Total	75	100	40	100	20	100	135	100

Most of the ever substance users [51(47.6%)] used substance once a day. It was found that nearly half of the ever substance users [47(44%)] practiced using substance for more than 4 years duration. Majority of the substance users cost 15–25 Ethiopian birr per day to use substances (Table 4).

**Table 4:** Pattern of ever substance use among substance users, COHMS, HU, Harar, Ethiopia, 2018 (*n* = 114)

Substance-using pattern		Chat		Cigarette		Alcohol	
		F	%	F	%	F	%
Frequency of substance use	Once a day	51	47.6	26	57.8	3	68
	Twice a day	34	31.8	11	56.7	1	2.3
	Once a week					1	2.3
	Twice a week					1	2.3
	3–4 times per week					9	20.5
	Some times	22	20.6	8	17.8	29	66
	Total	107	100	45	100	44	100
Duration of substance use	< 1 year	7	6.5	2	4.4		
	1–2 year	15	14.0	12	27.1	10	15.6
	2–4 year	38	35.5	16	33.3	7	6.3
	> 4 year	47	44	15	35.4	25	78.1
	Total	107	100	45	100	44	100
Amount of substance use per day (in birr)	5–15	62	58	45	100	10	22.3
	15–25	36	33.6	0	0	24	54.5
	25–35	9	8.4	0	0	3	6.8
	35–45	0	0	0	0	7	16
	Total	107	100	45	100	44	100

### Time of starting substance use and their plan when to stop

Regarding the initiation time of substance use, the majority [111(97.4%)] started using substances after 15 years of old. About 49% of the ever substance users started using substance before joining university whereas nearly 51% of them after joining university. Out of those 49% students who started substance use before joining university, the most majority [23(20.2%)] began substance use at preparatory school whereas from those students who started substance use after joining university (51%), being freshman (32.5%) was found to be a critical time to initiate substance use at university (Table 5). Among the total of 80 current substance users, 60(75%) have already planned to stop using the substance(s) of their choice whereas the rest 20(25%) not planned yet. From those 60(75%) who have planned to stop substance use, 30(37.5%) of them said that they would stop substance use practice after leaving the campus whereas the 22(27.5%) students plan to stop substance use in the near future.

**Table 5:** Time at which ever substance users started using substance, HU, Harar, Ethiopia, 2018 (*n* = 114)

Before joining University	Time	F	%
	Childhood	15	13.2
	Elementary school	11	9.6
	High school	7	6.1
	Preparatory school	23	20.2
	Sub-total	56	49.1
After joining University	During 1st year	37	32.5
	During 2nd year	14	12.3
	During 3rd year	5	4.4
	During 4th year	2	1.7
	During 5th year	0	0
	During 6th year	0	0
	Sub-total	58	50.9
Total		114	100

### Students’ reasons to use substances

A number of reasons have been listed by students to start substance use of which to keep alert while studying [80(29.6%)] was the leading reason enumerated by students. The others were to be free from psychological stress [63(23.3%)], peer pressure [35(13%)], role modeling of families and teachers [29(10.7%)], religious purpose [22(8.1%)], socialization purpose [21(7.8%) and lack of adequate recreational area [20(7.4%)].

### Correlation of undesirable health effects with ever substance use

Among all the 251 respondents, 230(91.6%) were aware of all the undesirable health effects of substance use. Out of 114 ever substance users, 31.6% of them had undesirable health effect that may be correlated with their substance use behavior. Anorexia [40 (35.1%)], insomnia [29 (25.4%)], depression [25 (22%)], gastritis [25 (22%)], dental caries [23 (20.2%)] and increased sexual activity [12 (10.5%)] were the major undesirable health effects reported by the students thought to result from their substance use (Table 6). Regarding sexual risk behavior among ever substance using students, 15 (68.2%) of the respondents had multiple sexual partners, 13 (59.1%) used condom irregularly, and 6 (27.2%) had sex with commercial sex workers. From the study, it was also resulted that among 114 ever substance users, 46(40.4%) ever had social and economic difficulties from their substance use. Among these, 43 (93.5%) had difficulties in covering monthly expenditure, 20 (43.5%) had an objection from family members due to financial inadequacy, 8 (18.6%) had falling injury and criminal acts whereas 7(16.3%) of whom felt stigma and discrimination.

**Table 6:** Frequency distribution of undesirable health effects that substance users ever had, COHMS, HU, Harar, 2018 (*n* = 114)

Undesirable health effect	F	%
Anorexia	40	35.1
Insomnia	29	25.4
Depression	25	22
Gastritis	25	22
Dental caries	23	20.2
Increased sexual activity	12	10.5
Libido	6	5.3
Week physical fitness	6	5.3
Easy vulnerability to several diseases	1	0.9

### Factors associated with substance use among students

In this study, based on the cross tabulation, sex, having sexual risk behavior, monthly income and family history of substance use were found to be significantly associated with substance use among students as evidenced by X^2^ values of 19.67, 28.99, 72.28 and 139.72 with *P*- value of 0.05 respectively. Students’ sex was one of the factors which was significantly associated with substance use in the study area. Male students were twenty times more likely to be substance user than female students (X^2^ = 19.67 at *P*- value of 0.05). Similarly, those students who have sexual risk behavior were twenty-nine times more likely to be substance user than students who had no sexual risk behavior (X^2^ = 28.99 at *P*- value of 0.05). Regarding monthly income and family history of substance use, students who had monthly income more than 540 Ethiopian birr were seventy-two times more likely to be substance user than students who earned a monthly income less than 540 Ethiopian birr (X^2^ = 72.28 at *P*- value of 0.05). Likewise, students who had a family history of substance use were one hundred forty times more likely to be substance user than students who had no family history of substance use (X^2^ = 139.72 at *P*- value of 0.05) (Table 7).

**Table 7:** Factors associated with ever substance use among health and medical science students of HU, Harar, Ethiopia, 2018 (*n* = 114)

		Ever substance user			X^2^ at *p* = 0.05 and d_f_ = 1 = 19.67
		Yes	No	Total	
Sex	Male	94	77	171	
	Female	20	60	80	
	Total	114	137	251	
		Ever substance user			X^2^ at *p* = 0.05 and d_f_ = 1 = 28.99
		Yes	No	Total	
Sexual Risk Behavior	Yes	22	0	22	
	No	92	137	229	
	Total	114	137	251	
		Ever substance user			X^2^at *p* = 0.05 And d_f_ = 1 = 72.28
		Yes	No	Total	
Average estimated monthly income	<540	21	99	120	
	≥ 540	93		131	
	Total	114	137	251	
		Ever substance user			X^2^ at *p* = 0.05 And d_f_ = 1 = 139.72
		Yes	No	Total	
Family history of using the same substance	Yes	68	8	76	
	No	46	129	175	
	Total	114	137	251	

## Discussion

One of the targets in the health goal of the sustainable development goals (SDGs) is to strengthen the prevention and treatment of substance use, including narcotic drug use and harmful use of alcohol. Therefore, this study is aimed to show the prevalence and associated factors of substance use in the study area after the launch of SDG by 2015 that involves the implementation of different preventive and therapeutic measures. Prevalence of ever substance use for at least one substance was found to be 45.5%. This finding is almost consistent with a study in many African universities which was 69.8% [6] but higher than Ethiopian studies at Mekelle University (21%), Debre markos Poly Technique College (14.1%), Hosana Health Science College (27%) and Jimma University (12.4%) [3, 18, 19, 21]. This discrepancy may be due to a smaller sample size that was considered for this study and differences in geographic location (khat is much more cultivated and marketed nearby this study area than elsewhere in Ethiopia). Moreover, organizational, physical and behavioral property variables of campuses including the type of residence, institutional size and campus community could be reasons to the variation. This study showed that 68(63.6%) of the ever chewers used to practice coffee drinking whereas 40(37.4%) of them cigarette smoking and 22(19.3%) alcohol drinking along with their chewing habit. On the other hand, the study conducted in Jimma students about khat chewing in 1994 witnessed that 57.9% of khat chewers did smoke cigarette and 44.3% consumed alcoholic beverages [19]. This shows that khat chewing was not practiced alone even about 20 years ago and it is still practiced along with these habits. From this study, it was resulted that most of the ever substance users accounting for 64.9% started the practice before joining university from whom 30(26.3%) began at preparatory school, 7(6.1%) at high school, 22(19.3%) at elementary school and 15(13.2%) started during childhood. Similarly, the study done up on 423 students of Debre Markos Poly Technique College showed 3.6% of the respondents started using substances when they were at elementary school, 3.56% at secondary school, 15.4% at preparatory school and 10.7% at the college level [3]. Therefore, both studies showed that the prevalence of substance use (to start the action) increases as age and educational level increases and becomes highest at preparatory schools and universities. In this study, it was found that majority of the ever substance users [80(70.2%)] started substance use to keep alert while studying, 63(55.3%) to get relieved of stress, 35(30.7%) due to peer pressure and 20(17.5%) for relaxing themselves with their friends whereas a research conducted among college students in Northwest Ethiopia revealed that the main reasons were to keep alert while studying, for relaxation with friends, peer pressure and for relieving stress each accounting for 129(47.4%), 160(76.4%), 70(23.9%) and 54(30.9%) respectively [17].This similarity in their reasons might be due to almost similar patterns of life at university. Many of the respondents knew the health risks of substance use; as a result, 60(77.5%) of the ever substance users had planned to stop the practice from whom 22(27.5%) planned to stop it in the near future, 7(8.75%) after graduation. Similarly, a study done among medical and other department students of Jimma University, Southwest Ethiopia showed that 68.2% of substance users had planned to stop using the substance in the near future or after leaving the campus [19]. This showed that most of the students use the substance for different conditions in the campus during campus life and have a plan to be withdrawn from the habit after being graduated or leaving the campus. From this study, out of 114 ever substance users, 22(19.3%) claimed to have sexual risk behavior due to their substance use. From these 22(19.3%) ever substance users with sexual risk behavior, 15 (68.2%) of them had multiple sexual partners, 13 (59.1%) used condom irregularly, and 6 (27.2%) had sex with commercial sex workers. However, a study conducted at Hosanna health science college, Southern Ethiopia, revealed that out of 423 participants, 313(74.0%) had sexual risk behavior of whom 157(50.5%) practiced sex with multiple sexual partners, 66(21.1%) used condom irregularly and 19(15.9%) practiced sex with female commercial sex workers in bars and brothel putting themselves at risk for HIV infection [21]. This significant discrepancy in the prevalence of sexual risk behavior might be due to the difference in cultural background between the two study areas. Furthermore, a smaller sample size of this study might have contributed to this variation.

## Conclusions

As compared to other studies, this study showed a higher magnitude of ever substance use and majority of whom are still using substance. This indicates the number of youngsters being involved in this practice is increasing despite the different preventive and therapeutic interventions that have been taken into action since 2015. Male sex, sexual risk behavior, average monthly income and family history of substance use were significantly associated with increased odds of ever substance use. The study also found that the leading reason for substance use was to keep alert while studying followed by psychological stress, peer pressure, role modeling of families and teachers, socialization purpose and lack of adequate recreational activities. Since all of these reasons are modifiable, every health preventive, promotive and rehabilitative action can be considered of paramount significance. Therefore, families and teachers should be role models in disliking substance use. Moreover, freshman students should be told and oriented of better stress coping mechanisms so that they can adapt themselves to the new university environment. Lastly, the authors would like to extend their recommendation to the college of health and medical science itself to expand more recreational activities so that students can prefer these services instead of substance use during their break time.

## Strength and limitation of the study

As this study focused on undergraduate health science students, the findings would be helpful to initiate effective substance use control programs in health science schools. Likewise, it can also be used as a blueprint to conduct an interventional study in the particular area. However, it was not possible to establish a temporal relationship between the exposure and outcome variable as this study design was a cross-sectional study. The result may not be representative of entire university students in Ethiopia due to a small sample size of the study.

## Supplementary information

**Additional file 1:** Questionnaire A structured questionnaire used for interviewing selected health and medical science students of HU, 2018.

## Data Availability

Data will be available upon request from the corresponding author.

## Abbreviation

AAU: Addis Ababa university
COHMS: College Of Health and Medical Science
Df: Degree of Freedom
EPHA: Ethiopian Public Health Association
HU: Haramaya University
JU: Jimma University
MLT: Medical Laboratory Technician
WHO: World Health Organization
