# Supplementary material for: Prevalence, associated factors and consequences of substance use among health and medical science students of Haramaya University, eastern Ethiopia, 2018: a cross-sectional study
Source: BMC Psychiatry. 2019 Nov 6;19:343. doi: 10.1186/s12888-019-2340-z (PMC6836499; doi:10.1186/s12888-019-2340-z)
Supplement: Supplementary file 1 — Additional file 1: Questionnaire A structured questionnaire used for interviewing selected health and medical science students of HU, 2018. [file 12888_2019_2340_MOESM1_ESM.rtf]

PART I: socio demographic information.
Sno	Question	Response	Skip rule	
1	Age	----(in completed years)		
2	Sex	A.	Male     □
B.	Female  □		
3	Religion	A.	Orthodox □
B.	Muslim   □
C.	Protestant □
D.	Catholic  □
E.	Other    □		
4	Ethnicity	A.	Oromo  □
B.	Amhara □
C.	Tigray   □
D.	Guraghe □
E.	Aderie  □
F.	Somalie □
G.	Other   □		
5	Department	A.	Nursing□
B.	Medicine□
C.	Health officer□
D.	Environmental health science□
E.	Mid-wifery□
F.	Pharmacy□
G.	Psychiatry□
H.	MLT□		

6	Class year	1st year□       4th year□
2nd  year□	 5th year□
3rd  year□	 6th year□
		

7	Monthly income	-----------(in Ethiopian birr)		

Part II: prevalence of substance abuse
1.	 Have you ever seen students who use substances in Harar COHMS?
A.	Yes                           B. NO
1.1.	If yes, which of the following substances you have seen being used ?
A.	 khat      B. cigarette      C. Alcohol      D. others (hashish,cannabis,cocaine,etc.)
2.	Have you yourself ever used any of the above substances?
A.	Yes                         
B.	No
2.1.	If yes, which one(s)? (You can use more than one answer)
A.	.khat  
B.	cigarette  
C.	Alcohol    
D.	Others (hashish, cocaine, cannabis,etc.)

A.	Khat related questions
Sno	Question	Response	Skip rule	
1	Have you ever chewed khat?	Yes□
No□	If “ No” go to only question 4	
1.1	If yes, at what age did you start chewing khat?	----(in year)		
2	For how long have you chewed khat?	Only once in life□
Less than one year□
1-2 years□
2-4 years□
>4 years□		
3	How often do you chew khat?	Once a day□
Twice aday□
Once a week□
Twice a week□
3-4 days per week□
Sometimes□		
4	Do your families have the habit of chewing?	Yes□
No□		
5	How much do you cost for chewing khat per day?	10-20 birr □
20-30 birr □
30-40 birr □
>40 birr   □		
6	When did you start chewing khat?	Before joining university □
After joining university □		
6.1	If before joining university, when?	During childhood    □
At elementary school□
At junior high school□
At preparatory school □		
6.2	If after joining university, when?	During 1st year□
During 2nd year□
During 3rd year□
During 4thyear□
During 5thyear□
During 6thyear□		
7	Have you chewed khat in this month?	Yes□
No□		
8	Are you able to easily access khat from outside the college and take it into your dormitory?	Yes□
No□		
9	What do you usually practice along with your khat chewing?	Alcohol drinking□
Cigarette smoking□
Coffee drinking□
Other(mention) -------		
10	Have you planned to stop khat chewing?	Yes □
No  □		
10.1	If yes, when?	Since now □
In the near future □
After leaving the college □
Other(mention) □		

B .Cigarette related questions
SNO	Question	Response
	Skip rule	
1	Have you ever smoked cigarette?	Yes □
No  □	If 'No' go to only question 4	
1.1	If yes, at what age did you start smoking cigarette?	
------- (in completed years)		
2	How long have you smoked?	Once in life□
Less than one year□
1-2 years□
2-4 years□
>4 years□		
3	How often do you smoke?	Once a day□
Twice a day□
Once a week□
Twice a week□
3-4 days per week□
Sometimes□
		
4	Do your families have the habit of smoking cigarette?	Yes □
No□
		
5	How much do you cost for smoking per day?	< 3 birr	□          9-12 birr□
3-6 birr	□           ≥12 birr□
6-9 birr□		
6	When did you start smoking cigarette?	Before joining university□
After joining university□		
6.1	If before joining university, when?	During childhood      □
At elementary school □
At junior high school □
At preparatory school□		
6.2	If after joining university, when?	During 1st year□
During 2ndyear□
During 3rd year□
During 4thyear□
During 5th year□
During 6th year□		
7	Have you smoked cigarette in this month?	Yes□
No □		
8	Are you able to easily access cigarette and smoke in the college?	Yes □
No □		
9	Have you planned to stop smoking?	Yes□
No□		
9.1	If yes, when?	Since now□
In the near future□
After leaving the college□
Other(mention)---------		

3.	Alcohol related questions
SNO	Question	Response	Skip rule	
1	Have you ever drunk alcohol?	Yes□
No□	If 'No' go to only question 4	
1.1	If yes, at   what age did you start drinking alcohol?	--(in completed year)		
2	For how long have you drunk alcohol?	Once in life □
Less than one year□
1-2 year□
2-4 year□
>4 year□		
3	How often do you drink alcohol?	Once a day□
Twice a day□
Once a week□
Twice a week□
3-4 days per week□
Sometimes□		
4	Do your families have the habit of drinking alcohol?	Yes□
No□		
5	How much do you cost for drinking alcohol per day?

	5-20 birr□
20-35 birr□
35-40 birr□
≥40 birr□		
6	When did you start drinking alcohol?	Beforejoining university□
After joining university□		
6.1	If before joining university, when?	During childhood□
At elementary school□
At junior high school□
At preparatory school□		
6.2	If after joining, university when?
	During 1styear□
During 2ndyear □
During 3 rdyear□
During 4th year□
During5th year□
During 6thyear□		
7	Have you drunk alcohol in this month?	Yes□
No□		
8	Are you able to easily access and drink alcohol outside the campus?	Yes□
No□		
9	Have you planned to stop drinking alcohol?	Yes□
No□		
10	If yes when?	Since now □
In the near future □
After leaving the college □
Other(mention) --------		

PART III: Reasons for using the substances (khat, cigarette, alcohol and others)
Sno	Question	Rsponse	Skip rule	
1	Why did you start using the substance of your choice?(you can give more than one reason)	To keep alert while studying □

To get relieved of psychological stress(tension)  □

To kill extra time due to lack of adequate recreational activities   □

Simply by seeing teachers and families using the same substance□

For socialization purpose   □

For religious purpose      □

Peer pressure □

Other(mention)□
	Skip this question, if you are not ever chewer, ever drinker and/ever smoker	
2	Who did energize you to practice using the substance of your choice?	Family□
Friend□
Other(mention)	Skip it if you are not ever chewer, ever smoker and/ever drinker	

Part IV: Undesirable health effect(s) correlated with substance use
1.	Have you ever had any undesirable health effect(s)as a consequence of your chewing, smoking and/drinking?
A.	 Yes □   
B.	 NO □
2.	If yes, which of the following? (You can encircle more than one answer if there is any)

A.	lung cancer
B.	constipation
C.	anorexia (loss of appetite)
D.	gastritis
E.	stained teeth (dental carries)
F.	difficulty to fall asleep (insomnia)
G.	Depression
H.	Change in sexual drive:  impotence (Loss of sexual interest) □
I.	Libido (Decreased sexual activity) □
J.	Increased sexual activity □
K.	Weak physical fitness
L.	Liver disease
M.	Cardio vascular diseases (hypertension, heart disease)
N.	Easy vulnerability to several disease
3. Are you aware of all the above health risks of substance use?
           A. yes □      B. No □
4.   Have you ever had any sexual risk behavioras a result of your substance using practice?
          A.Yes □           B.No □
4.1. If yes, which of the following? (You can encircle more than one answer if there is any)

         A. Multiple sexual partners
         B. Irregular condom use
C. committing sex with female commercial sex workers in bars and brothels
D. Other.

5. Have you ever had social and economic difficulties as a result of your chewing, smoking and/drinking?
         A. yes □    B. No □
5.1. If yes, which of the following? (You can encircle more than one choice if there is any)

      A. Difficulties in covering monthly expenditure

      B. Objection from family members due to inadequacy of finance
      C. Falling physical injuries and criminal acts (theft)
      D. stigma and discrimination because of undesirable behavior.
